# Automated Detection of Bone Fractures in Muscle X‐Ray Images Using Multiband‐Frequency Aware Deep Representation Learning

**DOI:** 10.1049/htl2.70021

**Published:** 2025-10-12

**Authors:** Rishab Kumar Pattnaik, Rajesh Kumar Tripathy, Haipeng Liu

**Affiliations:** ^1^ Department of Electrical and Electronics Engineering BITS‐Pilani Hyderabad India; ^2^ Centre for Intelligent Healthcare Coventry University Coventry UK

**Keywords:** accuracy, bone fracture, deep representation learning, MXR image, transfer learning, wavelet transform

## Abstract

The automated detection of bone fractures in muscle X‐ray (MXR) images using artificial intelligence is vital for successful treatment and better patient outcomes. This paper proposes the multiband‐frequency aware deep representation learning network (MFADRLN)‐based automated approach for detecting bone fractures in MXR images. The discrete wavelet transform‐based multiresolution analysis is utilised to evaluate subband images from the MXR image. Then, the deep representation learning (DRL) is applied to each subband of the MXR image, followed by feature concatenation, a dense layer, and a sigmoid layer for detecting bone fractures. The DRL branch for each subband mainly consists of the pre‐trained or frozen EfficientNetV2B2‐based block, a flattened layer, two successive dense‐batch normalisation (BN)‐dropout layer blocks, followed by the sigmoid layer for extracting multi‐band frequency‐aware features from MXR images. The MFADRLN model's importance is to capture the frequency‐specific and spatial information of the MXR image and obtain an improved feature representation for efficient detection of bone fractures. The publicly available musculoskeletal X‐ray image databases are used to evaluate the performance of the proposed MFADRLN‐based approach. The results reveal that the MFADRLN has obtained the accuracy and F1‐score values of 92.22% and 0.841, respectively, for detecting bone fractures. The proposed approach has demonstrated superior performance compared to the existing transfer learning techniques (ResNet50, EfficientNetV2B2, DenseNet201, MobileNetV2, InceptionV3, and XceptionNet), Vision transformer and swin transformer models to detect bone fractures in MXR images from the same database. The classification performance of the MFADRLN is compared with existing deep‐learning techniques for detecting bone fractures in MXR images.

## Graphical abstract

The automated detection of bone fractures from muscle X‐ray (MXR) images using artificial intelligence is vital for successful treatment and better patient outcomes. We proposed a novel multi‐frequency aware deep representation learning network (MFADRLN)‐based automated approach. The proposed model outperformed existing transfer learning models, providing a new option for reliable detection of bone fractures using MXR images.

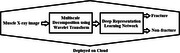

**Abbreviations**
BN: batch normalisation
MFADRLN: multiband‐frequency aware deep representation learning network
MXR: muscle X‐ray
TL: transfer learning

## 1 Introduction

Bone fractures are the most common musculoskeletal damages that occur due to traumas, accidents, sports injuries, and osteoporosis [1]. There is an increase in the number of patients with musculoskeletal damage globally and in India due to improper lifestyle and aging [2]. Accurately detecting bone fractures is essential for effective treatment and reducing the risk of complications. The muscle X‐ray (MXR) is the primary diagnostic test that orthopaedic specialists or radiologists use in the clinical standard for diagnosing bone fractures [1]. The visual inspection of the MXR image‐based manual approach has been used to detect bone fractures. This type of manual approach is time‐consuming and heavily dependent on the skills and experience of the orthopaedic specialists for detecting bone fractures in MXR images. Artificial intelligence (AI)‐based automated systems are helpful in the early detection of bone fractures by reducing human error and minimising the delay in the treatment using MXR images [3]. Such systems support radiologists and orthopaedic specialists by highlighting subtle changes in the morphological characteristics of the MXR images, which are often missed in the manual diagnosis procedure for detecting bone fractures [3]. The AI‐based systems use either machine learning (ML) or deep learning (DL)‐based algorithms to detect bone fractures in MXR images. The ML‐based approaches rely on steps such as manual extraction of features from MXR images using different image processing techniques and the feature selection strategy for obtaining better classification performance for detecting bone fractures [3]. On the other hand, DL‐based techniques reduce the need for feature engineering and automatically learn the hierarchical features at different layers from MXR images for accurate detection of bone fractures [3]. Some of the important challenges for the design of automated systems for the detection of bone fractures in MXR images are the quality of MXR images, false positives due to small or subtle fracture regions in MXR images, the limited availability of annotated MXR images for model training, the class imbalance issue of the model, and overfitting of the model. Therefore, it is interesting to develop new DL‐based automated techniques for the accurate detection of bone fractures in MXR images.

In recent years, different DL‐based methods have been reported for detecting bone fractures in MXR images [4] [3]. Ma and Luo [5] have a crack‐sensitive convolutional neural network (CNN) model for detecting bone fractures in MXR images. Wang et al. [6] have suggested a parallelNet, which consists of a two‐stage region‐based CNN for detecting bone fractures in MXR images. The hybrid approach using CNN and bidirectional long short‐term memory (BLSTM) has been utilised to detect bone fractures in MXR images [7]. The AlexNet and ResNet50‐based pre‐trained blocks are used to extract features, and BLSTM has been employed for classifying fracture and non‐fracture classes using MXR images [7]. Mishra et al. [8] have used the transfer learning (TL) blocks to extract features from MXR images and the support vector machine to detect bone fractures. Guan et al. [9] have used the vision transformer (ViT) to detect bone fractures in MXR images. Similarly, Selvaraj et al. [10] have suggested a hybrid model using the CNN and ViT to detect bone fractures with the input as MXR images. Jones et al. [11] have developed an ensemble DL framework comprising ten CNN models for the automated detection of bone fractures using musculoskeletal radiographs or MXR images. Aldhyani et al. [12] have formulated a hybrid framework using pre‐trained TL blocks, dilated convolution layers, attention layers, and dense layers for classifying bone fractures and non‐fractures using MXR images. Tahir et al. [13] have implemented the ensemble DL‐based framework by considering the decisions of different TL techniques, such as ResNet50, InceptionV3, MobileNetV2 and VGG16, to detect bone fractures in MXR images. They have also performed a comparative study with thirteen different TL methods for detecting bone fractures with input as the MXR images [13]. A multi‐modal approach based on the use of ViT and clinical bidirectional encoder representations from transformer (BERT) as feature extractors from MXR images and the patient's report, followed by the XGBoost classifier, has been used for detecting bone fractures in MXR images [14]. The performance of the existing DL‐based methods has been evaluated using MXR images from different datasets to detect bone fractures. The DL models show less classification performance across the datasets due to the variations in the patient demographic information, the quality of MXR images, and the protocols involved in recording MXR images [15]. The model trained using the MXR images from one dataset may fail to obtain better classification performance when tested using the MXR images from another dataset for detecting bone fractures. Furthermore, the class imbalance issues in some datasets can lead to biased predictions of the DL model for detecting bone fractures in MXR images. The existing DL‐based methods also lack interpretability, making it difficult for radiologists to believe the model's prediction without knowing the types of features extracted from MXR images for detecting bone fractures [16]. To address such limitations, a novel DL‐based approach that mainly focuses on improved feature extraction for obtaining higher accuracy is required, and it is interpretable for the automated detection of bone fractures in MXR images.

The multiband‐frequency or multi‐scale domain deep representation learning (DRL) and DL approaches have been used in different biomedical applications [17, 18, 19]. In such multiband‐frequency domain DRL models, the image decomposition method is first performed to obtain the subbands or components from the medical image. Then, the pre‐trained or TL blocks are utilised to get the local features for classifying different diseases [17]. The DRL models also have faster convergence during training due to fewer trainable parameters and better performance on small datasets [17]. The multiband‐frequency domain DRL model has not been investigated for detecting bone fractures in MXR images. The advantages of the multiband‐frequency domain DRL model are given as follows. The use of DWT in the initial stage helps to segregate the information of the MXR image into different frequency components, which is crucial for detecting small fracture regions. The low‐frequency component or approximation subband captures structural information, whereas the high‐frequency components (detail subbands) capture the edge and texture information in the MXR image. The DRL model uses the pre‐trained TL blocks on each subband to evaluate the local representations across different frequency bands and provides discriminative features for the detection of bone fractures in MXR images. The originality of this work is to develop a multiband‐frequency aware DRL network (MFADRLN) to detect bone fractures using the input as the MXR images. The salient contributions of this paper are given as follows:

- The two‐dimensional discrete wavelet transform (2DDWT)‐based multi‐resolution analysis technique is utilised to evaluate subbands from MXR images, enabling the multiband frequency‐specific feature‐based analysis to detect bone fractures.
- The frozen or pre‐trained EfficientNetV2B2‐based DRL network is applied to each subband to extract multiband‐frequency aware representations, enhancing the MFADRLN model's ability to capture complementary information across different subbands.
- The multi‐frequency aware feature representations from all four subbands are concatenated and processed through dense layers to detect bone fractures in MXR images.
- The classification outcome of the proposed MFADRLN is compared with various TL techniques, including Vision Transformer (ViT) and Swin Transformer (SwiT), to demonstrate its effectiveness in detecting bone fractures in MXR images.

The remaining sections of this paper are organised as follows: Section 2 describes the MXR databases used in this work in detail. The proposed multi‐frequency aware DRL model is described in Section 3. The results and the discussions of these results for this work are presented in Section 4. Finally, the conclusions of this paper are drawn in Section 5.

## 2 MXR Image Datasets

In this work, we have considered the MXR images from different datasets to evaluate the performance of the proposed MFADRLN for detecting bone fractures. The first dataset (Mandely dataset) contains 1211 and 1173 MXR images for simple fractures and comminuted fractures, respectively [20]. The Mandely dataset also contains 127 MXR images for the non‐fracture class. Similarly, the second dataset (FracAtlas dataset) consists of 4083 MXR images [21]. 1538, 2272, 338 and 349 hand, leg, hip and shoulder X‐ray images are given [21]. In the FracAtlas dataset, the MXR images include multiple fracture regions or locations. The 717 fractured MXR images comprise a total of 922 fracture regions. Among these, 437, 63, 63 and 263 hand, shoulder, hip and leg fracture regions are given in the FracAtlas dataset. Due to the presence of fractures in multiple regions, the FractAtlas dataset is valuable for developing AI‐based models for automatically detecting bone fractures in MXR images. While processing MXR images, we encountered a few MXR image files from both datasets that were not opened in the software‐based framework for developing the model to detect bone fractures. Therefore, we have identified those MXR images and excluded them from the datasets to train the proposed MFADRLN model for detecting bone fractures. After processing, the data distributions for both fracture and non‐fracture classes for the Mandely dataset and FracAtlas dataset cases are shown in Table 1. We have considered 717 and 3337 MXR images for fracture and non‐fracture classes from the FracAtlas dataset. Similarly, 2048 and 127 MXR images for fracture and non‐fracture classes are used from the Mandely dataset.

**TABLE 1:** Data distribution for fracture and non‐fracture classes for Mandely and FracAtlas datasets.

Dataset	Fracture	Non‐fracture	Data selection	Fracture	Non‐fracture	Total
**Mendeley** [20]	2048	127	**Training**	1990	2494	4484
**FracAtlas** [21]	717	3337	**Validation**	222	277	499
**Total**	2765	3464	**Test**	553	693	1246

## 3 Proposed Method

**FIGURE 1:**
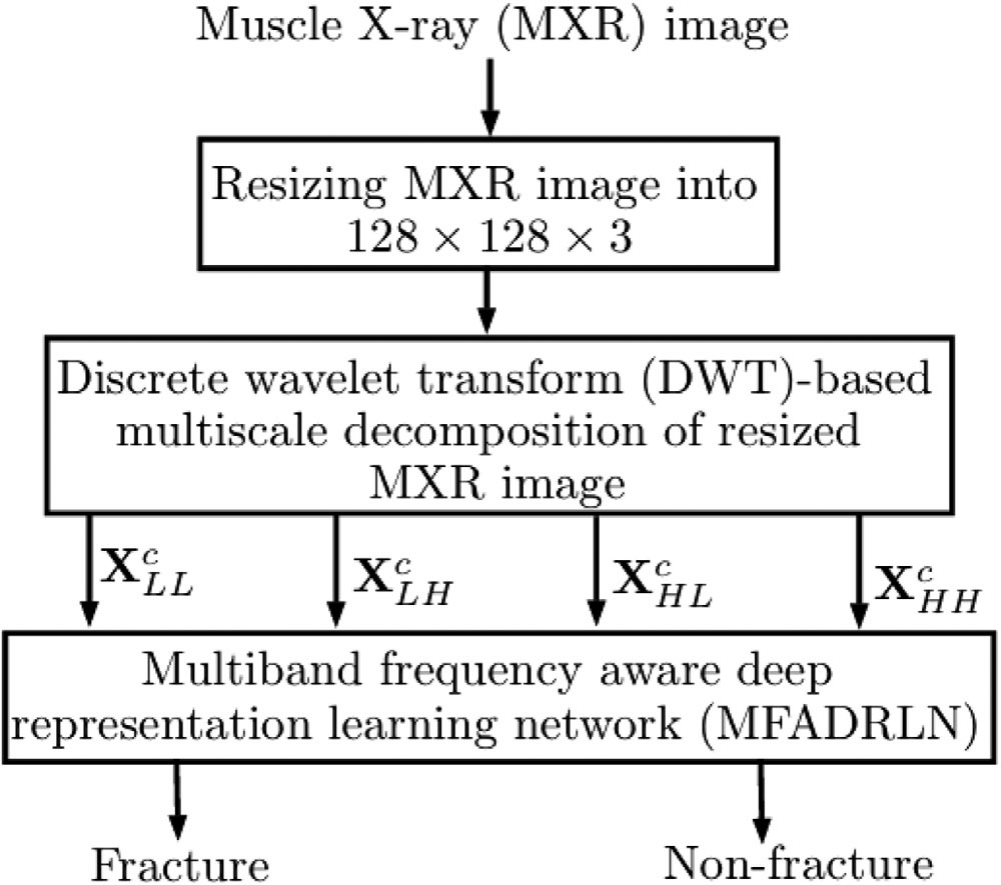
Overall system architecture of the proposed approach for detection of bone fractures in MXR images.

The overall system architecture of the proposed approach for detecting bone fractures in MXR images is depicted in Figure 1. The proposed approach consists of the resizing of the MXR image, multiscale decomposition of the MXR image, and the MFADRLN model for detecting bone fractures. We have resized the MXR images of both fracture and non‐fracture classes into 128×128×3. After obtaining the resized MXR image, the multiscale decomposition is performed to evaluate the sub‐band images.

### 3.1 Multiscale Decomposition of MXR Image

**FIGURE 2:**
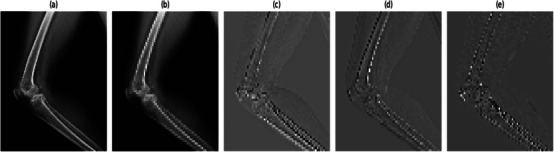
(a) Original MXR image for non‐fracture class. (b) LL‐subband of the MXR image for the non‐fracture class. (c) LH‐subband of the MXR image for the non‐fracture class. (d) HL‐subband of the MXR image for the non‐fracture class. (e) HH‐subband of the MXR image for the non‐fracture class.

**FIGURE 3:**
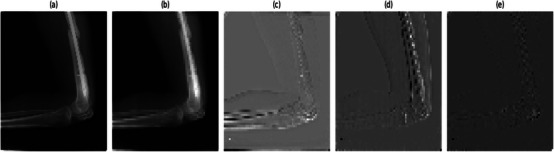
(a) Original MXR image for fracture class. (b) LL‐subband of the MXR image for the fracture class. (c) LH‐subband of the MXR image for the fracture class. (d) HL‐subband of the MXR image for the fracture class. (e) HH‐subband of the MXR image for the fracture class.

In this work, the 2DDWT is utilised for the multiresolution analysis of MXR images. For the MXR image, $X^{c}={\left[x{\left(i,j\right)}^{c}\right]}_{i=0,j=0}^{M,N}$, the size of c ^th^ channel is given as M×N. The mathematical expressions to evaluate LL, LH, HL, and HH subbands for c ^th^ channel using 2DDWT are given as follows [22, 23]:

(1) $${\text{LL}}^{c}\left(m,n\right)=\sum_{i=0}^{M-1}\sum_{j=0}^{N-1}X^{c}\left(i,j\right)\phi \left(2m-j\right)\phi \left(2n-i\right)$$

(2) $${\text{LH}}^{c}\left(m,n\right)=\sum_{i=0}^{M-1}\sum_{j=0}^{N-1}X^{c}\left(i,j\right)\phi \left(2m-j\right)\psi \left(2n-i\right)$$

(3) $${\text{HL}}^{c}\left(m,n\right)=\sum_{i=0}^{M-1}\sum_{j=0}^{N-1}X^{c}\left(i,j\right)\psi \left(2m-j\right)\phi \left(2n-i\right)$$

(4) $${\text{HH}}^{c}\left(m,n\right)=\sum_{i=0}^{M-1}\sum_{j=0}^{N-1}X^{c}\left(i,j\right)\psi \left(2m-j\right)\psi \left(2n-i\right)$$

where ϕ(k) and ψ(k) are the low‐pass and high‐pass analysis filters. In 2DDWT, first, the row‐wise filtering followed by downsampling, and then the column‐wise filtering followed by downsampling are performed to evaluate the wavelet coefficients in different subbands [22, 23]. The size of each subband (LL, LH, HL and HH) for c ^th^ channel is $\frac{M}{2}\times \frac{N}{2}$. The subband images for c ^th^ channel ($X_{\text{LL}}^{c}={\left[X_{\text{LL}}^{c}\left(i,j\right)\right]}_{i=0,j=0}^{M,N}$, $X_{\text{LH}}^{c}={\left[X_{\text{LH}}^{c}\left(i,j\right)\right]}_{i=0,j=0}^{M,N}$, $X_{\text{HL}}^{c}={\left[X_{\text{HL}}^{c}\left(i,j\right)\right]}_{i=0,j=0}^{M,N}$ and $X_{\text{HH}}^{c}={\left[X_{\text{HH}}^{c}\left(i,j\right)\right]}_{i=0,j=0}^{M,N}$) are evaluated based on the inverse 2DDWT‐based reconstruction and these are given as follows [22, 23]:

(5) $$X_{\text{LL}}^{c}\left(i,j\right)=\sum_{m=0}^{\frac{M}{2}-1}\sum_{n=0}^{\frac{N}{2}-1}{\text{LL}}^{c}\left(m,n\right)\tilde{\phi }\left(2m-j\right)\tilde{\phi }\left(2n-i\right)$$

(6) $$X_{\text{LH}}^{c}\left(i,j\right)=\sum_{m=0}^{\frac{M}{2}-1}\sum_{n=0}^{\frac{N}{2}-1}{\text{LH}}^{c}\left(m,n\right)\tilde{\phi }\left(2m-j\right)\tilde{\psi }\left(2n-i\right)$$

(7) $$X_{\text{HL}}^{c}\left(i,j\right)=\sum_{m=0}^{\frac{M}{2}-1}\sum_{n=0}^{\frac{N}{2}-1}{\text{HL}}^{c}\left(m,n\right)\tilde{\psi }\left(2m-j\right)\tilde{\phi }\left(2n-i\right)$$

(8) $$X_{\text{HH}}^{c}\left(i,j\right)=\sum_{m=0}^{\frac{M}{2}-1}\sum_{n=0}^{\frac{N}{2}-1}{\text{HH}}^{c}\left(m,n\right)\tilde{\psi }\left(2m-j\right)\tilde{\psi }\left(2n-i\right)$$

where $\tilde{\phi }\left(k\right)=\phi \left(-k\right)$ and $\tilde{\psi }\left(k\right)=\psi \left(-k\right)$ are the low‐pass and high‐pass synthesis filters. Each of the subband images was evaluated from the MXR image using 2DDWT and inverse 2DDWT for c ^th^ channel is of size M×N. The four subband image tensors obtained from the multiscale analysis of MXR images using 2DDWT and inverse 2DDWT for all three channels are given as $X_{\text{LL}}$, $X_{\text{LH}}$, $X_{\text{HL}}$ and $X_{\text{HH}}$, respectively. In this work, the size of the MXR image is 128×128×3. Thus, the size of each sub‐image tensor is the same as that of the original MXR image. The plots of the original MXR image and the LL, LH, HL, and HH subband image tensors for the non‐fracture class are depicted in Figure 2a and Figure 2b–e, respectively. Similarly, for the fracture class, the plots of the original MXR image and the LL, LH, HL, and HH subband image tensors are displayed in Figure 3a and Figure 3b–e, respectively. The information of the MXR image has been segregated into different subbands based on the frequency range for fracture and non‐fracture classes. Such local information of MXR images is helpful for better classification of fractures and non‐fractures. In this work, the MFADRLN, which consists of the deep representation learning (DRL)‐EfficientNetV2B2 block applied on each subband image tensor, a dense layer, is suggested to detect bone fractures using the MXR images. We have also considered two‐dimensional stationary wavelet transform (2DSWT) [24] and singular value decomposition (SVD) [17] for decomposing the MXR images. The basis functions, such as Haar, Daubechies (Db4, Db5, and Db8), are used for 2DDWT and 2D SWT cases. The optimal basis function for analysing MXR images is evaluated based on the classification performance of the MFADRLN model to detect bone fractures. For the SVD case, we have considered the grouping of eigentriples (1–5 for subband1, 6–15 for subband2, 16–40 for subband3, and 41–128 for subband4) [17] to evaluate the modes or subbands from the MXR images. We have selected the optimal decomposition technique out of 2DDWT, 2DSWT and SVD for the multiresolution analysis of MXR images based on the classification performance of the MFADRLN model.

### 3.2 MFADRLN Model

**FIGURE 4:**
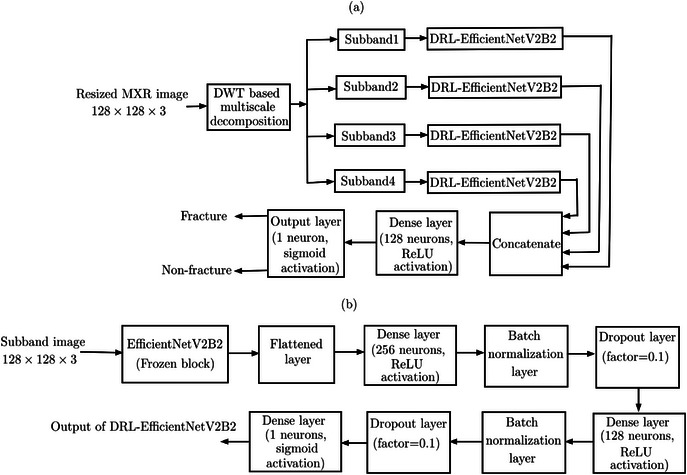
(a) Block diagram of the proposed MFADRLN for detecting bone fractures in MXR images. (b) The layers of the DRL‐EfficientNetV2B2 block for extracting multiscale or multiband‐frequency‐based features from MXR images.

The flowchart of the proposed MFADRLN model architecture for detecting bone fractures in MXR images is depicted in Figure 4a. The MFADRLN model consists of DRL‐EfficientNetV2B2 blocks, which are applied to all four subbands to evaluate the probability scores. The architecture of the DRL‐EfficientNetV2B2 is depicted in Figure 4b. The input to the DRL‐EfficientNetV2B2 block is the subband image tensor with size 128×128×3. The DRL‐EfficientNetV2B2 consists of the frozen or pre‐trained EfficientNetV2B2 block, flattened layer, dense layers, batch normalisation (BN) layers, and dropout layers, respectively. The size of the tensor obtained after the frozen EfficientNetV2B2 block is 4×4×1408. The output of the DRL‐EfficientNetV2B2 block is the probability value or the score obtained using the sigmoid activation function, as depicted in Figure 4b. For each subband of the MXR image in Figure 4a, the probability or score is evaluated using DRL‐EfficientNetV2B2. The probability values for subband1 (LL), subband2 (LH), subband3 (HL) and subband4 (HH) subband image tensors are denoted as $P_{\text{LL}}$, $P_{\text{LH}}$, $P_{\text{HL}}$ and $P_{\text{HH}}$, respectively. The subband probability vector, P is created by appending the probability values of each subband evaluated from the MXR image, and it is given as follows:

(9) $$P=[P_{\text{LL}},P_{\text{LH}},P_{\text{HL}},P_{\text{HH}}]$$

After obtaining the subband probability vector, we have considered a dense layer and the output layer (sigmoid activation function) for detecting bone fractures. The dense layer consists of 128 neurons with rectified linear unit (ReLU) activation function. The cost function used for the training of the MFADRLN model is the binary cross‐entropy function, and it is given as follows [25]:

(10) $$L=\frac{-1}{m}\sum_{i=1}^{m}\left[y^{i}\log\left(h^{i}\right)+\left(1-y^{i}\right)\log\left(1-h^{i}\right)\right]$$

where m is the number of instances or batches. $y^{i}$ and $h^{i}$ are the actual output and hypothesis for i ^th^ instance or MXR image. The Adam optimiser is used to obtain the optimal weight values in the dense layers of the proposed MFADRLN model. The training, validation, and test sets (MXR images) of the proposed MFADRLN model are evaluated using hold‐out validation and ten‐fold cross‐validation (CV) strategies [26]. For hold‐out validation, 72%, 8%, and 20% of the MXR images from the total set (6229 MXR images) are used for the training, validation and test sets of the MFADRLN network to detect bone fractures. The number of muscle X‐ray images in the training, validation, and test sets is mentioned in Table 1 for fracture and non‐fracture classes. The hyperparameters, such as the initial learning rate, batch size and number of epochs, are selected as 0.001, 32 and 50, respectively. These hyperparameters are chosen based on a grid search based on the highest value of the accuracy of the validation set [26]. In this work, we have used BN, dropout, and early stopping to prevent the overfitting of the MIFDNN model for detecting bone fractures in MXR images. The BN and dropout layers are used in the DRL‐EfficientNetV2B2 block part for each subband, as shown in Figure 4b. The BN layer is a regulariser by reducing the covariance shift and stabilising the learning process [25]. Similarly, the dropout layer randomly deactivates the neurons in the training of the MIFDNN model. It helps to learn robust and discriminative features for accurately detecting bone fractures in MXR images. Early stopping is used to train the MIFDNN model to halt training when validation accuracy is not improving for five consecutive epochs [25]. These three strategies effectively overcome the overfitting issues in the MIFDNN model during training for detecting bone fractures in MXR images. Moreover, we have also used various TL techniques such as (VGG16 [27], ResNet50 [28], EfficientNetV2B2 [29], Densenet201 [30], MobileNetV2 [31], InceptionV3 [32], and XceptionNet [33]), vision transformer (ViT) pretrained [34] and swin transformer (SWiT) pre‐trained [35] models, and compared the classification performance of these networks with the proposed MFADRLN model for detecting bone fractures in MXR images. For the pre‐trained ViT, the base model (ViT‐B/16) [36], which has a path size of 16×16 and the number of transformer layers of 12, is used. Similarly, for the pre‐trained SWiT [35], the base model with a window size of 7×7, path size as 4×4, and number of transformer blocks as 12 is used. The last layer is removed for the pre‐trained models, and a new output layer with a sigmoid activation function is utilised. The fine‐tuning is performed only for the last layer weight and bias values for the learning, ViT‐B/16, and SWiT (pre‐trained) models. The hyperparameters are considered the same for all the TL, ViT‐B/16 and SWiT (pre‐trained) models as those of the MFADRLN model to detect bone fractures in MXR images. Moreover, the custom ViT and SWiT models are also implemented and compared with the proposed MFADRLN model to detect bone fractures in MXR images. For the first custom ViT model (custom ViT1), we have considered four transformer encoder layers, four attention heads, the projection dimension, 64, and the number of MLP heads as [128,64], respectively. Similarly, for the second custom ViT model (custom ViT2), eight transformer encoder layers, eight attention heads, the projection dimension as 64, and the number of MLP head units as [128,64] are considered. For the first custom SWiT model (custom SWiT1), the patch size, the projection dimension, the number of heads, the number of transformer layers, the MLP head units, and the window size are considered as 8×8, 64, 4, 4, [128,64], and 4×4, respectively. Likewise, for the second custom SWiT model (custom SWiT2), we have considered the patch size as 8×8, the projection dimension as 64, the number of heads as eight, number of transformer layers as eight, the MLP head units as [128,64] and the window size as 4×4, respectively. The classification performance of the proposed MFADRLN model, TL, and transformer models is evaluated using the test muscle X‐ray images with the measures such as precision, recall, accuracy, F1‐score and Kappa score, respectively [37]. These metrics are calculated from the confusion matrix obtained based on the actual test output and the predicted test output of the MFADRLN model for detecting bone fractures. The receiver operating characteristics (ROC) curve and the precision–recall curve (PRC) [38] are also utilised in this work for evaluating the performance of the classifiers for detecting bone fractures in MXR images.

## 4 Results and Discussion

In this section, we have shown the classification results of the proposed MFADRLN model, the comparison of the MFADRLN model with TL and existing approaches, and the deployment of the MFADRLN model on WebApp for the detection of bone fractures in MXR images. The advantages and the shortcomings of the work are also mentioned in Section 4.3.

### 4.1 Classification Results of the Proposed MFADRLN Model

**FIGURE 5:**
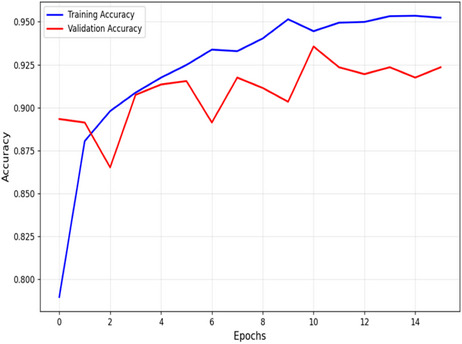
Accuracy versus epoch plot of the proposed MFADRLN model for detecting bone fractures in MXR images.

**FIGURE 6:**
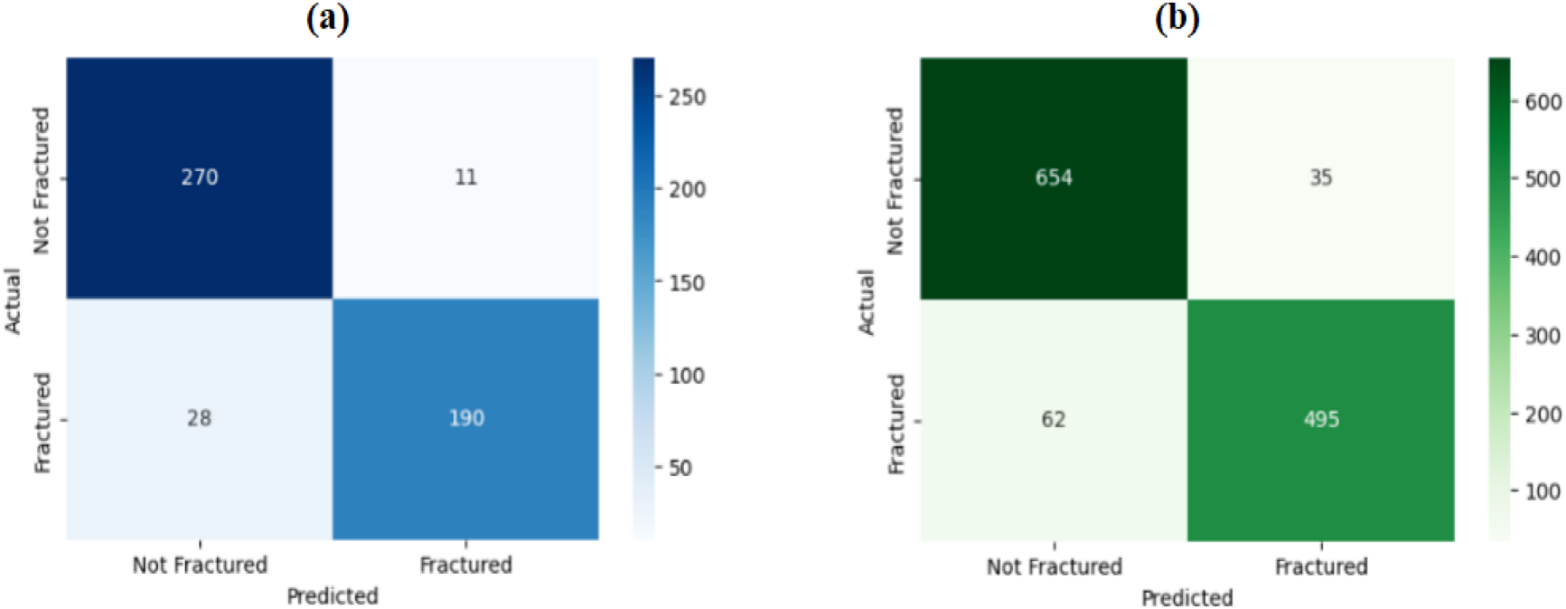
(a) Confusion matrix of the MFADRLN model using MXR images from the validation set. (b) Confusion matrix of the MFADRLN model using MXR images from the test set.

**FIGURE 7:**
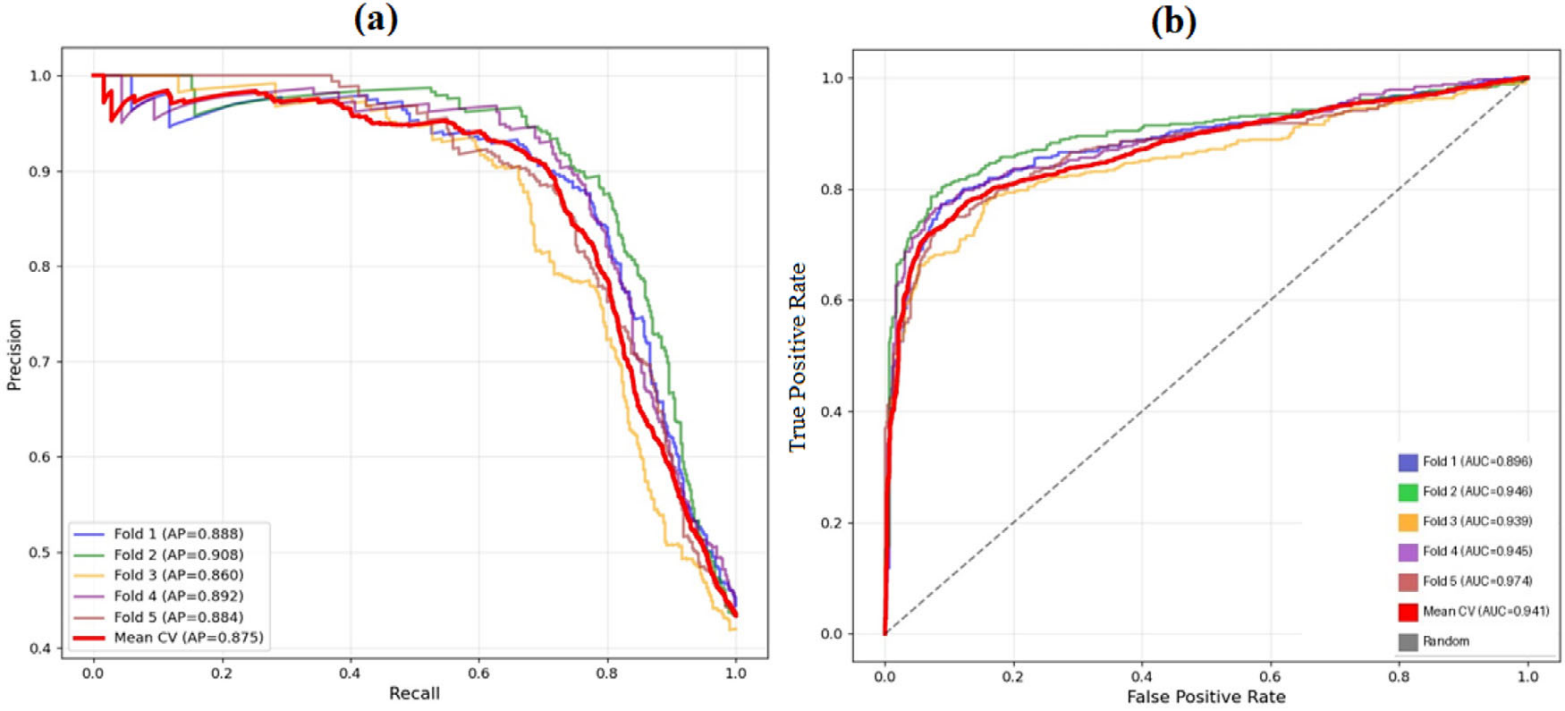
(a) Precision–recall curve of the MFADRLN model evaluated for fivefold CV. (b) ROC curve of the MFADRLN model evaluated for fivefold CV.

The accuracy versus epoch plot of the proposed MFADRLN model for the training and validation data (MXR images) is shown in Figure 5. The difference of 2.5% between the training and validation accuracy values suggests that the proposed MFADRLN model provides a better generalisation for the automated detection of bone fractures in MXR images. The confusion matrices of the proposed MFADRLN model evaluated using the MXR images from the validation set and test sets are depicted in Figure 6a,b, respectively. The validation accuracy and test accuracy values of the MFADRLN model are obtained as 92.18% and 92.21%, respectively. The classification results of the proposed MFADRLN models with different pre‐trained blocks for detecting bone fractures in MXR images are shown in Table 2. It is evident that the MFADRLN with EfficientNetV2B2 as pre‐trained blocks has demonstrated the accuracy, precision, recall, F1 score, and Kappa score values of 92.22%, 93.40%, 88.87%, 91.08%, and 0.8418, respectively, to detect bone fractures in MXR images. The MFADRLN with ResNet50 and MFADRLN with XceptionNet models have obtained accuracy values of 86.52% and 87.16%, respectively. The other MFADRLN models have obtained lower classification accuracy values than MFADRLN with EfficientNetV2B2 models for detecting bone fractures in MXR images. We have also evaluated the classification results of the MFADRLN with the EfficientNetV2B2 model for detecting bone fractures in MXR images with a fivefold CV, which are depicted in Table 3. The proposed MFADRLN model has obtained accuracy values of more than 90%, for fold2, fold3, fold4, and fold5, respectively. For the fold5 case, the Kappa score value of 0.80 has been reported by the MFDRLN model, which is the highest compared to other folds for detecting bone fractures in MXR images. It is observed that the MFADRLN with the EfficientNetV2B2 model has obtained the average accuracy and average precision values of more than 90% for detecting bone fractures in MXR images. Similarly, the average recall, average F1‐score, and average Kappa score values of more than 80% are reported using the proposed MFADRLN model. The precision–recall (PR) and receiver operating characteristic (ROC) curves of the proposed MFADRLN model is shown in Figure 7a,b, respectively. The mean area under the PR curve (AUPRC) and mean area under the ROC curve (AURC) values of 0.875 and 0.941 are obtained using the MFADRLN model for fivefold CV for detecting bone fractures in MXR images. The MFADRLN model has demonstrated more than 90% accuracy values for both validation cases to classify fracture and non‐fracture classes using MXR images.

**TABLE 2:** Classification results of MFADRLN‐based models for detecting bone fractures in MXR images with hold‐out validation.

MFADRLN models	Accuracy (%)	Precision (%)	Recall (%)	F1‐score (%)	Kappa
**MFADRLN‐VGG16**	69.74%	99.41%	30.88%	47.12%	0.333
**MFADRLN‐ResNet50**	86.52%	100.00%	68.12%	81.04%	0.711
**MFADRLN‐EfficientNetV2B2**	**92.22**%	**93.40**%	**88.87**%	**91.08**%	**0.841**
**MFADRLN‐DenseNet201**	81.38%	96.80%	60.11%	74.16%	0.608
**MFADRLN‐MobileNetV2**	83.31%	97.24%	66.32%	78.86%	0.658
**MFADRLN‐InceptionV3**	73.03%	63.86%	86.22%	73.38%	0.472
**MFADRLN‐XceptionNet**	87.16%	96.77%	72.57%	82.94%	0.729

**TABLE 3:** Classification results of the MFADRLN model for detecting bone fractures in MXR images with five‐fold CV.

Folds	Accuracy (%)	Precision (%)	Recall (%)	F1‐score (%)	Kappa
Fold 1	89.37	98.65	77.62	86.88	0.781
Fold 2	90.26	98.39	79.01	87.64	0.797
Fold 3	90.10	96.15	81.05	87.95	0.796
Fold 4	90.18	93.32	83.82	88.31	0.798
Fold 5	90.66	97.80	78.90	87.34	0.800
μ±σ	90.11±0.42	96.86±1.97	80.08±2.17	87.62±0.49	0.795±0.006

**FIGURE 8:**
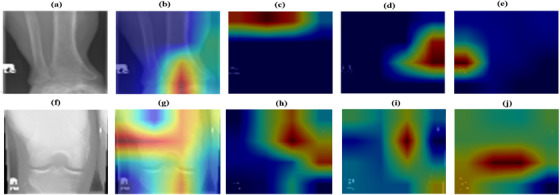
(a) Original MXR image for fracture class. (b) Grad‐CAM plot evaluated for the LL‐subband image of the MXR image. (c) Grad‐CAM plot evaluated for the LH‐subband image of the MXR image. (d) Grad‐CAM plot evaluated for the HL‐subband image of the MXR image. (e) Grad‐CAM plot evaluated for the HH‐subband image of the MXR image. (f) Original MXR image for fracture class. (g) Grad‐CAM plot evaluated for the LL‐subband image of the MXR image. (h) Grad‐CAM plot evaluated for the LH‐subband image of the MXR image. (i) Grad‐CAM plot evaluated for the HL‐subband image of the MXR image. (j) Grad‐CAM plot evaluated for the HH‐subband image of the MXR image.

The classification results of MFADRLN with the DRL‐EfficientNetV2B2 model are evaluated using different subband image selection strategies of the MXR images, and these results are shown in Table 4. It is evident that when LL and HL‐subband images of the MXR image are considered, the MFADRLN model has obtained an accuracy value of 92.05% for detecting bone fractures in MXR images. Similarly, when LL, LH, and HL subband images are utilised, the MFADRLN model has produced an accuracy value of 90.93%, lower than the LL and HL‐subband images‐based MFADRLN model for detecting bone fractures. The HH‐subband image‐based MFADRLN has obtained the lowest accuracy value of 83.23% for detecting bone fractures in MXR images. When all subbands are used, the MFADRLN model has demonstrated the highest accuracy compared to other subband combinations in MFADRLN models for the automated detection of bone fractures in MXR images. The computational time of the proposed MFADRLN model using the selected subbands of the MXR image for detecting bone fracture is depicted in Table 4. It is observed that the computational times of the proposed MFADRLN models are relatively similar when two, three and four sub‐bands of MXR images are used to detect bone fractures. The computational time may not increase linearly even after adding more subbands as the input to the MIFDRLN model, as modern central processing units (CPUs) process the information in parallel. Due to this reason, the computational time is found to be relatively similar for MFADRLN models with different subband‐based selection strategies for detecting bone fractures in MXR images. Similarly, the classification performance of MFADRLN is evaluated using different image decomposition techniques, such as 2DDWT, 2DSWT and SVD, respectively, and the results are shown in Table 5. It is observed that the MFADRLN model with 2DDWT‐based subbands of MXR images has obtained higher classification accuracy than the 2DSWT and SVD‐based subbands. The 2DDWT‐based multi‐resolution analysis approach effectively captures the local information of the MXR image in different subband images. Hence, the MFADRLN model has produced higher accuracy using the subband images obtained using 2DDWT than other image decomposition methods, such as 2DSWT and SVD methods. Moreover, we have also evaluated the classification performance of the MFADRLN model using 2DDWT‐based subbands of MXR images with different basis functions (Haar, db4, db5, db8) for detecting bone fractures. These results are shown in Table 6. It is observed that the MFADRL model with 2DDWT‐based subbands evaluated from MXR images with the ‘Haar’ basis function has demonstrated higher accuracy than other basis functions (Db4, Db5, and Db6) for detecting bone fractures. To better understand the prediction of the proposed MFADRLN model, we have evaluated the gradient‐weighted class activation mapping (Grad‐CAM) plot for each subband image of the MXR images for fracture and non‐fracture classes. The original MXR images for fracture and non‐fracture classes are displayed in Figure 8a,f, respectively. The Grad‐CAM plots evaluated for the MFADRLN model for LL, LH, HL, and HH‐subband cases for fracture and non‐fracture classes are depicted in Figure 8b–e, g–j, respectively. The proposed MFADRLN model has considered the features from each subband of MXR images for improved bone fracture detection. The LL‐subband‐based Grad‐CAM of the proposed MFADRLN model has highlighted the broader region around the irregular structure in the bone contour or fracture part. Similarly, for the non‐fracture class, the attention of the MFADRLN model is evenly distributed across the bone structure as seen in Figure 8g. The Grad‐Cam plots for HL, LH and HH subbands capture the directional discontinuities and fine details of MXR images for the automated classification of fracture and non‐fracture classes.

**TABLE 4:** Subband‐wise classification results of MFADRLN‐EfficientNetV2B2 model for detecting bone fractures in MXR images with hold‐out validation.

Subband selection	Accuracy (%)	Precision (%)	Recall (%)	F1 score (%)	Kappa	Computational time in sec (μ±σ)
LL	91.09	94.40	85.05	89.48	0.817	0.241±0.044
LH	88.20	86.38	86.22	86.30	0.759	0.236±0.021
HL	91.73	94.89	86.25	90.36	0.831	0.248±0.062
HH	83.23	76.96	89.46	82.74	0.666	0.238±0.066
LL and LH	91.17	92.92	87.39	90.07	0.821	0.256±0.054
LL and HL	92.05	94.35	87.89	91.01	0.839	0.242±0.051
LL and HH	91.49	94.40	85.21	89.57	0.824	0.238±0.044
LH and HL	90.13	93.61	81.19	86.96	0.790	0.217±0.017
LH and HH	90.77	90.46	87.94	89.18	0.811	0.219±0.019
HL and HH	91.17	92.66	87.39	89.95	0.820	0.249±0.056
LL, LH and HL	90.93	93.98	84.36	88.91	0.812	0.251±0.052
LL, LH and HH	91.33	95.83	83.94	89.49	0.821	0.223±0.039
LL, HL and HH	91.33	93.79	86.91	90.22	0.824	0.247±0.027
LH, HL and HH	91.41	92.16	87.52	89.78	0.823	0.239±0.036
All four subbands	**92.22**%	**93.40**%	**88.87**%	**91.08**%	**0.841**	0.251±0.060

**TABLE 5:** Classification results of MFADRLN model with different multi‐resolution analysis methods (2DDWT, 2DSWT and SVD) for detecting bone fractures in MXR images with hold‐out validation.

Model selection	Accuracy	Precision	Recall	F1 score	Kappa
MFADRLN with 2DDWT‐based subbands	92.22%	93.40%	88.87%	91.08%	0.841
MFADRLN with 2DSWT‐based subbands	90.20%	91.37%	88.39%	89.86%	0.803
MFADRLN with SVD‐based subbands	87.90%	86.34%	90.28%	88.26%	0.757

**TABLE 6:** Classification results of MFADRLN model with 2DDWT‐based subbands of MXR images with different basis functions (haar, db4, db5, db8) for detecting bone fractures with hold‐out validation.

Basis functions	Accuracy	Precision	Recall	F1 score	Kappa
**Haar**	**92.22%**	**93.40%**	**88.87%**	**91.08%**	**0.8418**
Db4	89.73%	97.34%	79.10%	87.28%	0.788
Db5	91.41%	96.45%	82.70%	89.05%	0.820
Db8	91.25%	96.97%	82.50%	89.15%	0.819

### 4.2 Comparison With TL Techniques and Existing Approaches

**FIGURE 9:**
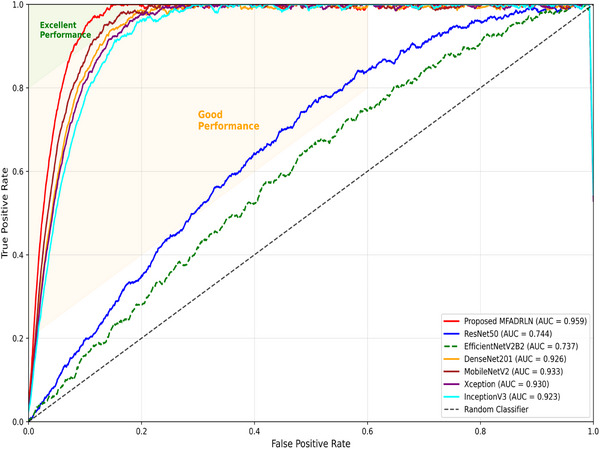
Comparison of ROC curves of different models for detecting bone fractures in MXR images.

We have compared the classification performance of the proposed MFADRLN with custom ViT and SwiT models in Table 7 to detect bone fractures in MXR images. It is observed that the custom ViT models with 4 and 8 transformer encoder layers have obtained accuracy values of 86.49% and 86.57%, respectively. Similarly, the custom SwiT models with 4 and 8 transformer encoder layers have produced accuracy values of 87.44% and 87.12% for detecting bone fractures in MXR images. The proposed MFADRLN has obtained higher accuracy, kappa, precision, recall and F1‐score values than the custom transformer models to detect bone fractures in MXR images. Furthermore, we have also compared the classification metrics of the MFADRLN model with various TL techniques and pre‐trained transformer models in Table 8 for detecting bone fractures in MXR images. The TL techniques, such as the ResNet50 [28], MobileNetV2 [31], InceptionV3 [32], XceptionNet [33], DenseNet201 [30] and EfficientNetV2B2 [29], have obtained accuracy values less than the proposed MFADRLN model for classifying fracture versus non‐fracture classes using MXR images. The pre‐trained ViT [36] and pre‐trained SwiT [35] base models have demonstrated the overall accuracy values of 84.42% and 87.84%, respectively. The MFADRLN model has obtained superior classification performance compared to the TL, ViT and SwiT models for detecting bone fractures using the MXR images. In Figure 9, we have compared the ROC curves of the proposed MFADRLN model with TL techniques for detecting bone fractures in MXR images. The area under the curve (AUC) values of the proposed MFADRLN model, transformers and TL techniques are depicted in Table 8. It is evident that the AUC value of the proposed MFADRLN model is higher than that of TL and transformer models for detecting bone fractures in MXR images. The TL and transformer‐based methods reported in Table 8 have used the MXR image directly for detecting bone fractures. The proposed MFADRLN model captures information on MXR images in different subbands or frequency levels. The DRL block applied to each subband of the MXR image has evaluated discriminative frequency‐specific representations at multiple scales. These discriminative features are concatenated and further processed in the dense layers for obtaining higher accuracy for detecting bone fractures. Hence, the proposed MFADRLN model has demonstrated higher classification performance in detecting bone fractures in MXR images.

**TABLE 7:** Comparison of the classification performance of the proposed MFADRLN model with different custom transformer architectures for detecting bone fractures in MXR images.

Transformer models	Accuracy	Precision	Recall	F1 score	Kappa
Custom ViT1	86.49%	89.35%	78.24%	83.43%	0.721
Custom ViT2	86.57%	88.16%	80.78%	84.31%	0.726
Custom SWiT1	87.44%	94.52%	75.55%	83.98%	0.738
Custom SWiT2	87.12%	91.65%	77.68%	84.09%	0.734
Proposed MFADRLN model	**92.22**%	**93.40**%	**88.87**%	**91.08**%	**0.841**

**TABLE 8:** Comparison with different TL and transformer‐based techniques to detect bone fractures in MXR images.

Model selection	Accuracy	Precision	Recall	F1 score	Kappa	AUC
ResNet50 [28]	85.22%	86.64%	86.33%	85.94%	0.764	0.744
EfficientNetV2B2 [29]	88.14%	89.75%	89.15%	88.92%	0.768	0.737
DenseNet201 [30]	84.57%	87.11%	86.37%	85.73%	0.695	0.926
MobileNetV2 [31]	84.57%	87.71%	86.75%	85.11%	0.698	0.933
InceptionV3 [32]	88.79%	89.48%	89.17%	89.39%	0.770	0.923
XceptionNet [33]	84.57%	87.35%	86.43%	85.24%	0.692	0.930
ViT B/16 (Pre‐trained) [36]	84.42%	88.84%	74.69%	81.15%	0.680	0.776
SWiT Base (Pre‐trained) [35]	87.84%	85.82%	85.98%	85.90%	0.7521	0.795
Proposed MFADRLN model	**92.22**%	**93.40**%	**88.87**%	**91.08**%	**0.841**	0.959

The classification results obtained using the proposed MFADRLN model have been compared with existing deep learning techniques in Table 9 for detecting bone fractures in MXR images. In [39], authors have used DenseNet169 and InceptionResNetV2‐based TL techniques for detecting bone fractures in MXR images and obtained the overall accuracy values of 84% and 86%, respectively. Similarly, in [39], authors have obtained an accuracy value of 78% using the VGG16‐based TL for detecting bone fractures. The histogram equalisation followed by the modified architecture‐based deep learning models, such as modified VGG16 and modified InceptionV3, demonstrated the overall accuracy values of less than 85% to detect bone fractures. The proposed MFADRLN model has demonstrated higher accuracy and other performance measures for detecting bone fractures than DenseNet169, VGG16, InceptionResNetV2, and modified deep learning‐based models.

**TABLE 9:** Performance comparison of the proposed MFADRLN model with various existing methods to detect bone fractures in MXR images.

Methods used	Accuracy (%)	Precision (%)	Recall (%)	F1 Score (%)
VGG16‐based TL [39]	78	77	77	78
DenseNet169‐based TL [40]	84	85	84	83
InceptionResNetV2‐based TL [40]	86	83	84	87
Histogram equalisation and modified InceptionV3 [13]	81	81	81	81
Histogram equalisation and modified VGG16 [13]	82	83	83	82
Proposed MFADRLN model	**92.22**	**93.40**	**88.87**	**91.08**

### 4.3 MFADRLN Model Deployment: Advantages and Limitations

**FIGURE 10:**
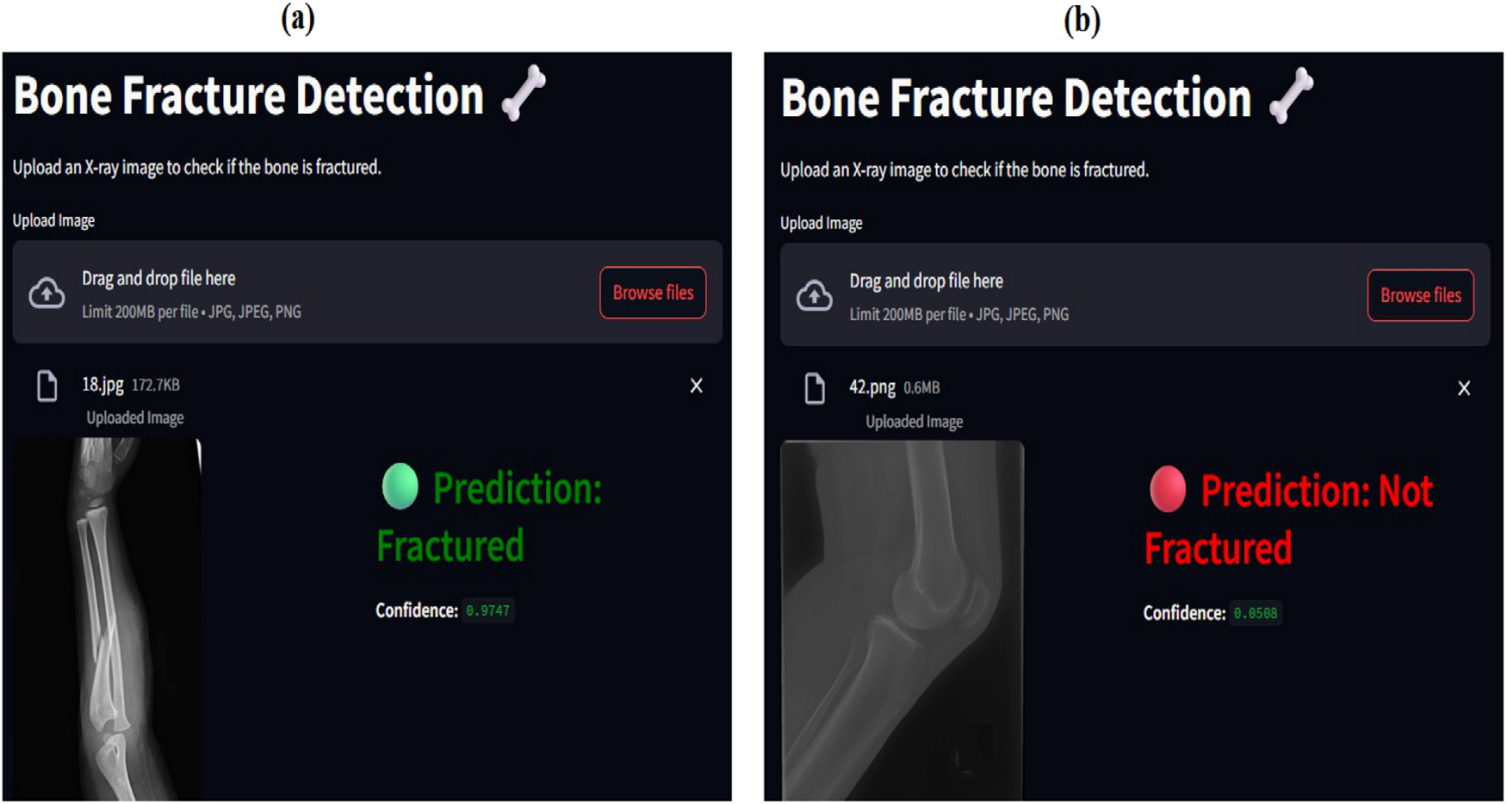
(a) WebApp‐based deployment of the proposed MFADRLN model for detecting fracture class using the MXR image. (b) WebApp‐based deployment of the proposed MFADRLN model for detecting non‐fracture class using the MXR image.

The proposed MFADRLN model is deployed in a web application (webApp) for Internet of Things (IoT)‐enabled automated detection of bone fractures in MXR images. The streamlit cloud‐based framework has been used for creating the webApp [41]. The trained MFADRLN model and the Python code in the testing phase are deployed in the streamlit cloud for designing the webApp. The webApp takes the input as the MXR image and predicts fracture or non‐fracture classes. The predictions of fracture and non‐fracture classes using the MFADRLN models on the webapp with input as MXR images are displayed in Figure 10a,b, respectively. The proposed MFADRLN model deployed on webApp has successfully predicted fracture and non‐fracture classes with confidence scores or probability values 0.97 and 0.05 using MXR images. The average time taken on the webApp for uploading the MXR image and predicting bone fracture is 12.01 s. The webApp‐based deployment of the MFADRLN offers several advantages, such as remote accessibility, real‐time diagnosis, and platform independence for detecting bone fractures in MXR images. The webApp‐based deployment of the proposed MFADRLN model has shortcomings, such as requiring stable internet connectivity to run the application. The privacy and the security of the patient data (MXR images) are important in webApp‐based deployment of an AI model for healthcare applications. Hence, the encryption of patient data and model parameters is required for developing webApp‐based healthcare systems. The uploading of high‐resolution MXR images into the webApp requires more time. Hence, the latency of the cloud‐based framework increases for real‐time detection of bone fractures in MXR images. Hence, the lightweight versions of deep learning models and their implementation on embedded devices like microcontrollers and field programmable gate array [42] can be investigated for detecting bone fractures in MXR images. The proposed MFADRLN model has shown the difference in the accuracy values when evaluated using separate datasets. Hence, the federated learning algorithms, which allow the training of deep learning models with different hospital datasets (MXR images) without sharing the patient data [43], can be incorporated to detect bone fractures in MXR images. Integrating electronic health records (EHRs), such as patient information like medical history, age, gender, previous bone fracture‐related injuries and usage of medicines, along with imaging modality, will be helpful to enhance the diagnostic accuracy for detecting bone fractures. The large language models such as BERT [44] with input as EHRs and deep‐learning‐based processing of MXR images can be used to create multimodal AI systems for automated detection of bone fractures.

## 5 Conclusion

In this study, we presented the MFADRLN‐based model for detecting bone fractures in MXR images. The multiscale domain subband images of the MXR image are evaluated using 2DDWT. A DRL framework based on the pre‐trained EfficientNetV2B2 and a few dense layers has been utilised on each subband image of the MXR image for detecting bone fractures. The results have demonstrated that the proposed MFADRLN has achieved an accuracy value of 92.22%, indicating a potential automated AI‐driven approach to assist radiologists in detecting bone fractures in MXR images. The proposed approach demonstrated superior classification accuracy compared to various TL techniques in detecting bone fractures in MXR images. The MFADRLN model also outperformed the classification performance of the transformer models, such as ViT and SwiT models, in detecting bone fractures. We deployed the MFADRLN model on a web app that allows healthcare providers to directly upload the musculoskeletal X‐ray image for real‐time detection of bone fractures. Future work will focus on developing deep learning‐based models for detecting a broader range of fracture types using MXR images.

## Author Contributions

**Rishabh Kumar Pattnaik**: conceptualisation, methodology, software, validation, writing – original draft. **Rajesh Kumar Tripathy**: investigation, resources, software, supervision, writing – review and editing. **Haipeng Liu**: investigation, supervision, validation, writing –review and editing.

## Conflicts of Interest

The authors declare no conflicts of interest.

## Data Availability

The data that support the findings of this study are openly available in [Mandely and figshare] at [https://data.mendeley.com/datasets/8d9kn57pdj/1, https://figshare.com/articles/dataset/The_dataset/22363012], reference numbers [20, 21].
